# Does Mental Well-Being Protect against Self-Harm Thoughts and Behaviors during Adolescence? A Six-Month Prospective Investigation

**DOI:** 10.3390/ijerph17186771

**Published:** 2020-09-17

**Authors:** Kirsten Russell, Susan Rasmussen, Simon C. Hunter

**Affiliations:** 1 School of Psychological Sciences and Health, University of Strathclyde, Glasgow G1 1QE, UK; kirsten.russell@strath.ac.uk; 2 Department of Psychology, Glasgow Caledonian University, Glasgow G4 0BA, UK; Simon.Hunter@gcu.ac.uk; 3 Faculty of Education, University of Western Australia, Perth, WA 6009, Australia

**Keywords:** self-harm, adolescence, defeat, entrapment, mental well-being, IMV

## Abstract

Mental well-being protects against the emergence of suicidal thoughts. However, it is not clear whether these findings extend to self-harm thoughts and behaviors irrespective of intent during adolescence—or why this relationship exists. The current study aimed to test predictions—informed by the integrated motivational–volitional (IMV) model of suicide—concerning the role of perceived defeat and entrapment within the link between mental well-being and self-harm risk. Young people (*n* = 573) from secondary schools across Scotland completed an anonymous self-report survey at two time points, six months apart, that assessed mental well-being, self-harm thoughts and behaviors, depressive symptomology and feelings of defeat and entrapment. Mental well-being was associated with reduced defeat and entrapment (internal and external) and a decrease in the likelihood that a young person would engage in self-harm thoughts and behaviors. The relationship between mental well-being and thoughts of self-harm was mediated by perceptions of defeat and entrapment (internal and external). Mental well-being was indirectly related to self-harm behaviors via decreased feelings of defeat and internal (but not external) entrapment. Taken together, these findings provide novel insights into the psychological processes linking mental well-being and self-harm risk and highlight the importance of incorporating the promotion of mental well-being within future prevention and early intervention efforts.

## 1. Introduction

Self-harm, defined as “self-injury or self-poisoning irrespective of the apparent purpose of the act” [1,2], first emerges as a significant public health problem during adolescence [3,4]. Evidence suggests that an increasing number of young people are harming themselves [5]. This is concerning as self-harm is indicative of intolerable psychological distress and young people who harm themselves are at significantly greater risk of suicide in the future (regardless of the intent underpinning these acts) [6,7]. Responding effectively to self-harm is therefore a key target for suicide prevention efforts. A fundamental component in reducing self-harm during adolescence is the identification of factors that increase or protect against self-harm risk and clarifying the theoretical underpinnings of these relationships.

It has been well-established that self-harm is the end-product of a complex and multifaceted pathway characterized by the interaction of biologic, genetic, environmental and psychological factors [8,9]. Historically, most research investigating self-harm risk during adolescence has focused on factors thought to increase the likelihood that a young person will think about or engage in self-harm (i.e., risk factors). Research on protective factors has been more limited [4,9]. Furthermore, research examining factors that protect against self-harm risk is rarely conducted through the lens of psychological theory. Examining the potential role of the theoretically salient psychological factors that may underpin these relationships is necessary if we are to understand why these factors reduce self-harm risk in young people. Since risk and protective factors are more likely to occur in combination than in isolation, it has been recognized that there is a need to develop more sophisticated explanatory models of self-harm that can help to conceptualize the complex interplay between risk and protective factors [8,10,11]. Research of this nature will aid in the identification of possible intervention opportunities within the pathway to self-harm. One model that is well-placed to facilitate this work is the integrated-motivational-volitional model [8,12].

### 1.1. Integrated Motivational-Volitional Model of Suicidal Behavior (IMV)

**Figure 1:**
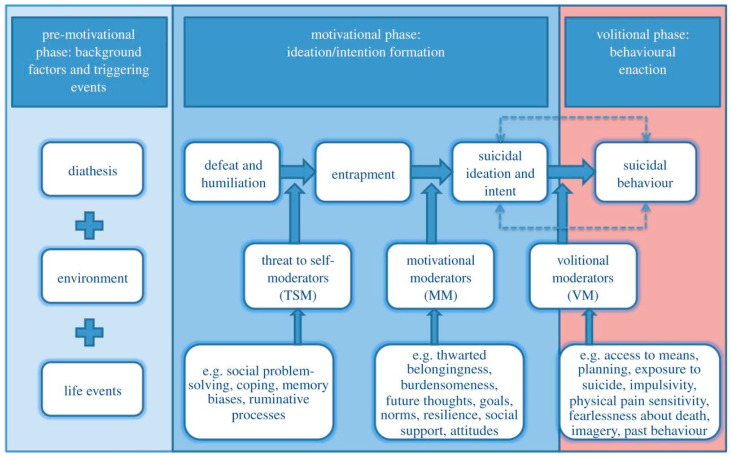
Visual representation of the integrated motivation–volitional model of suicidal behavior (IMV) [12].

The IMV is a tri-partite framework that maps out a clear pathway towards self-harm (Figure 1). Although the model was developed with suicidal behavior in mind, it can be applied to any self-harm thoughts and behavior (regardless of intent) [12,13]. The IMV describes how a multitude of factors interact and contribute to the development of self-harm thoughts (motivational phase), as well as to the transition from thoughts to acts of self-harm (volitional phase). At its core, the framework hypothesizes that perceptions of defeat and entrapment are key drivers of the intention to self-harm. More specifically, the model hypothesizes that when an individual perceives themselves to be trapped by internal and/or external factors in their life, they are more likely to develop self-harm thoughts. This intention to self-harm emerges because engaging in self-harmful behaviors is seen as the salient solution to escaping their thoughts, feelings and/or life circumstances. Feelings of entrapment are thought to be triggered by perceptions of defeat/humiliation, which are often associated with a range of background and triggering factors including stress (within the pre-motivational phase). The transition from defeat to entrapment is not inevitable and the likelihood of this progression can be facilitated or obstructed depending on the presence of factors known as threat-to-self moderators. Similarly, the transition from entrapment to self-harm thoughts can be strengthened or attenuated in the presence or absence of motivational moderators.

There is growing empirical support for the pathways and processes described in the IMV [12,13]. A substantial body of evidence supports the hypothesized role of defeat and entrapment as part of the psychological pathways that give rise to self-harm [14,15,16,17] and for the influence of threat-to-self and motivational moderators. While the IMV model provides one of the most comprehensive accounts to date of the processes involved in the development of self-harm thoughts and behaviors, it is still relatively new and, therefore, not exhaustive. Further, few studies have investigated the hypothesized pathways of the IMV in young people, despite adolescents being a high-risk group [18,19,20]. Identifying the factors associated with reduced risk of self-harm in young people and why, is key to understanding and preventing self-harm in this population [6,21]. As a result, there is a need to investigate the role of novel and modifiable protective factors within the context of the IMV. Mental well-being has received increasing international interest in recent years and is a likely, potential candidate in this regard. However, its potential application to self-harm risk is not yet fully understood.

### 1.2. Mental Well-Being During Adolescence

The World Health Organization [22] (p. 12) defines mental health as: ‘a state of well-being in which every individual realizes his or her own potential, can cope with the normal stresses of life, can work productively and fruitfully and is able to make a contribution to her or his community.’ This definition recognizes that mental health goes beyond a simple absence of mental illness. Mental well-being is a broad and complex construct that comprises two dimensions, namely how we feel (hedonic) and how we function psychologically and socially (eudemonic) [23,24,25]. It is the combination of these aspects that contribute to a young person being mentally healthy (i.e., feeling good and functioning well) [26,27]. Greater mental well-being appears to protect against a range of negative health, social and psychological outcomes [27,28,29]. Due to growing awareness of its public health impact the promotion of mental well-being during adolescence is becoming an international priority [30,31].

### 1.3. Does Increased Mental Well-Being Protect Against Self-Harm Risk During Adolescence?

While research has demonstrated that mental well-being protects against the development and maintenance of subsequent suicidal ideation in adults [32,33,34], it remains to be seen whether these prospective findings extend to adolescents. To our knowledge, only one study has investigated the relationship between mental well-being and self-harm risk in adolescents. Morey et al. [35] reported that adolescents with a history of self-harm were more likely to report lower levels of mental well-being. However, since that study was cross-sectional, prospective research is necessary to clarify the nature of the relationship and if mental well-being protects against self-harm risk more broadly (i.e., irrespective of intent) over time. In addition, the psychological processes driving the link between mental well-being and self-harm risk during adolescence are not known. Understanding these will be critical for developing effective and empirically informed interventions. As a result, there is a need to investigate the link within the context of self-harm theory. While mental well-being is not specifically highlighted within the IMV model, it may be hypothesized that it presents a potentially modifiable factor within different areas of this theoretical framework. Preliminary evidence suggests that mental well-being buffers the impact of entrapment on suicidal ideation in adults, thereby qualifying it as a motivational moderator based on the assertions of the IMV [36]. Given that adolescence is a high-risk developmental period for the onset and maintenance of self-harm, it is important that investigations seek to establish whether these findings extend to young people.

Within the first phase of the IMV (the pre-motivational phase) it is proposed that individuals may possess pre-existing vulnerability factors that pre-dispose them to respond adversely to stressors. The interaction between background factors and stressful circumstances is hypothesized to increase the likelihood that the individual will develop thoughts of self-harm via increased perceptions of defeat and entrapment within the motivational phase [12]. Mental well-being has been shown to interact with stressful life events, such that those with higher levels of mental well-being are less likely to develop suicidal ideation in response to elevated levels of stress [37]. As a result, it is possible that greater mental well-being may protect against risk of intentional self-injury and self-poisoning in young people by reducing perceptions of defeat and entrapment and buffering against the emergence of one’s intention to self-harm. It could therefore be hypothesized that mental well-being may fit as a pre-existing background protective factor within the pre-motivational phase. Given that depression (a disorder characterized by depressed mood and a loss of interest and/or pleasure) is associated with poorer mental well-being and increased risk of self-harm thoughts and behaviors [29], it is important to account for the influence of young people’s experiences of depressive symptoms when examining the role of mental well-being in self-harmful pathways.

### 1.4. The Current Study

**Figure 2:**
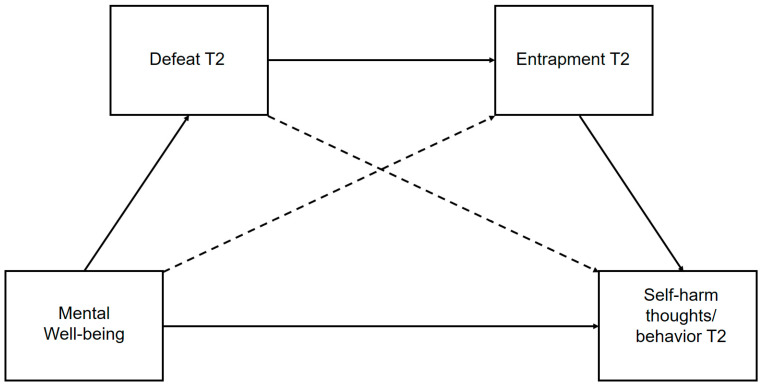
Predicted serial multiple mediation pathway (non-dashed lines) for association between mental well-being and subsequent self-harm thoughts, via defeat and entrapment. Predicted multiple mediation pathway highlighted in non-dashed lines. “T2” indicates data collected at the second time-point.

A limited body of research has investigated the link between mental well-being and self-harm risk. Clarifying the extent and nature of the interactions among these psychological factors may be important for future advances in self-harm intervention and prevention science. Therefore, the aims of the current study were fourfold:

1. To examine whether mental well-being protects against subsequent self-harm thoughts and behaviors;
2. To determine if mental well-being is associated with subsequent perceptions of defeat, internal entrapment and external entrapment;
3. To test hypothesized multistep pathways derived from the IMV that link mental well-being (as a potential pre-motivational factor) and subsequent self-harm thoughts and behaviors, via perceptions of defeat and entrapment (Figure 2);
4. To establish whether mental well-being moderates the relationship between entrapment and subsequent self-harm thoughts.

Four hypotheses were generated. First, based on the findings of previous cross-sectional research, it was hypothesized that mental well-being would be negatively associated with self-harm thoughts or behaviors during the six month follow-up period. Second, it was expected that mental well-being would be negatively associated with the three motivational factors (defeat, internal entrapment and external entrapment). We investigated both internal and external entrapment because recent work suggests that entrapment is best conceptualized as bidimensional and that each accounts for unique variance in adjustment indices [38,39]. Third, it was hypothesized that the prospective association between mental well-being and one’s intention to engage in self-harm would operate via a multi-stage indirect pathway. Specifically, it was predicted that mental well-being would be negatively associated with defeat; that defeat would in turn be positively associated with perceptions of entrapment (internal and external); and that entrapment would be positively related to self-harm thoughts and behaviors. Fourth, it was predicted that mental well-being would moderate the relationship between adolescents’ perceptions of entrapment and subsequent self-harm thoughts.

## 2. Materials and Methods

### 2.1. Participants

At baseline (T1: June 2015), we recruited 1045 adolescents (52.8% female) from 21 mainstream schools across Scotland. We requested permission from all local education authorities (*n* = 32) to contact their secondary schools and invite them to participate in the current study. Where approval was granted (*n* = 8), all mainstream schools within that authority were contacted. We recruited 28% of the target schools to this study. This is consistent with previous research investigating self-harm in adolescents [40]. Ages ranged from 15–17 years (M = 15.35, SD = 0.68). In terms of ethnicity, 97.2% of the sample was White. This is in line with the most recent Scottish census data (2011). There was representation from both urban (*n* = 16) and rural (*n* = 5) schools.

Of the initial sample, 54.8% (*n* = 573) completed measures at both time points and all analyses were constrained to this subsample. Reasons for non-participation at 6-month follow up (T2) included absence due to sickness or holidays, engagement in alternative activities and truancy (*n* = 305). Further, one school withdrew their participation between T1 and T2 (*n* = 167). Using t-tests and chi-squared tests, it was determined that those who completed the measures at 6-month follow up were similar to those who did not in terms of gender, mental well-being and self-harm thoughts (all *p*-values > 0.05). However, adolescents who did not complete measures at T2 reported more depressive symptoms at T1 (*t* (1045) =3.70, *p <* 0.001, Cohen’s *d* = 0.02) and were more likely to have a history of engaging in self-harm behavior (*X*^2^ (1) = 5.65, *p =* 0.018, Phi = 0.02). These differences were small in magnitude.

### 2.2. Measures

Demographic factors: Information on age, gender and ethnicity was collected to characterize the study sample.

Mental Well-being: The short version Warwick-Edinburgh mental well-being scale (SWEMWBS) [25] comprises 7 positively worded items that relate to different aspects of positive mental health. The scale has five response categories ranging from 1 (“none of the time) to 5 (“all of the time”). Higher scores indicate more positive mental well-being. The measure’s validity and reliability have been established in diverse populations, including secondary school pupils in the UK (aged 13 to 16) [41]. Mental well-being data were collected at both time points. Internal consistency was shown to be good in the current sample (T1: *α =* 0.87).

Depressive symptomology: The 7-item depression subscale of the hospital anxiety and depression scale (HADS) [42] is a valid and reliable measure of depressive symptomology, which is frequently used in community settings [19,43,44]. Participants are asked to indicate the extent to which they have experienced depressive symptoms in the past week on a Likert scale ranging from 0–3 and total scores are calculated by taking a sum of all responses. Data on depressive symptomology were collected at both time points. This measure was validated for use in adolescent samples and internal consistency was shown to be good in the present sample (T1: *α =* 0.86). Research has demonstrated that both mental well-being and self-harm are linked to depression. As a result, the HADS was included in the current study so that severity of depression could be adjusted for within our statistical analyses.

Thoughts of self-harm: History of self-harm thoughts was assessed using a single item, “Have you ever thought about taking an overdose or trying to harm yourself, but not actually done so?” Participants were asked to provide a binary response (yes/no). This measure has been used to assess self-harm thoughts in a range of school-based surveys across Europe [40,45,46]. At T2, participants were asked to respond to the same item, but were instructed to consider only the period that had elapsed since completing the T1 survey. As such, we were able to determine whether respondents had thought about self-harm, for the first time ever, during the period between T1 and T2.

Self-harm behavior: Acts of self-harm were assessed using one question taken from the child and adolescent self-harm in Europe (CASE) survey [45]. Adolescents were initially asked if they had ever deliberately taken an overdose (e.g., of pills or other medication) or tried to harm themselves in some other way (e.g., cutting themselves). Those who reported having engaged in self-harm were asked to think about their last act of self-harm and to describe in their own words (in as much detail as they felt comfortable with) how they had harmed themselves on that occasion. We asked young people to provide a description of their most recent experience of self-harm so that we were able to determine whether they met the CASE definition of self-harm: “an act with a non-fatal outcome in which an individual deliberately did one or more of the following: initiated behavior (e.g., self-cutting, jumping from a height), which they intended to cause self-harm; ingested a substance in excess of the prescribed or generally recognized therapeutic dose; ingested a recreational or illicit drug that was an act the person regarded as self-harm; ingested a non-ingestible substance or object” [45] (p. 28). Participants were asked about their history of self-harm at baseline (T1). During the follow-up (T2) assessment (six months later) they were asked if they had engaged in self-harm since participating in the first survey. As a result, we were able to establish if adolescents had intentionally harmed themselves during the follow-up period.

### 2.3. Procedure

Ethical approval was obtained from the University Ethics Committee (UEC16/47) at the lead author’s institution. Once permission was received from a hierarchy of gatekeepers (i.e., local education authorities, school gatekeepers and parents/guardians), young people were invited to take part in the study. All participants provided informed consent and pupils were aware of their right to withdraw their participation.

Respondents completed an anonymous self-report survey at two time-points, six-months apart within a school setting. In order to reinforce the private and confidential nature of the survey, young people answered questions under exam conditions (i.e., independently and in silence), the order of questions was counterbalanced, and participants sealed their completed questionnaires in an envelope before being returned to the researcher. Respondents generated a six-digit unique reference code by completing a series of questions that required alphanumeric responses at both time points. This allowed the research team to match response at follow-up, while maintaining the anonymity of the pupils who were participating. All young people were debriefed and provided with an information sheet that contained contact details for a range of local physical and mental health support services. Participants received no incentive for completing the survey.

### 2.4. Data Analytic Strategy

IBM SPSS Statistics 25 for Windows (IBM Corp, Armonk, NY, USA) was used to conduct all statistical analyses. Inspection of histograms revealed skewness across all independent variables. Accordingly, medians and interquartile ranges were calculated. Initially, Spearman correlational analyses were conducted to allow for a preliminary examination of the relationships between all variables assessed in the study. Given that self-harm engagement groups (self-harm thoughts vs. no self-harm thoughts and self-harm behaviors vs. no self-harm behavior) were unequal and that the assumption of homogeneity was violated for depressive symptomology, bootstrapping was applied to all analyses. Bootstrapping is a nonparametric resampling technique in which repeated samples are taken from the original dataset to estimate the sampling distribution of a statistic. In this study, analyses were based on 5000 sample bootstrap replications.

To address the first aim of the current investigation, two binary logistic regressions were conducted to determine whether mental well-being predicts subsequent self-harm thoughts and/or self-harm behavior. The reference category for these analyses were young people who had not thought about harming themselves and young people who had not harmed themselves during the six-month follow-up period, respectively.

To determine whether mental well-being is associated with motivational factors within the IMV, a series of three multivariate regressions were conducted. The outcome variables for each of these analyses were defeat, internal entrapment and external entrapment. All outcome variables were assessed at T2.

Next, a serial multiple mediation pathway was tested using Model 6 of the PROCESS algorithm for SPSS [47] whereby the relationship between mental well-being and prospective self-harm thoughts was mediated by perceptions of defeat and entrapment at T2. Two separate mediational models were run. In the first model, the predictor variable was mental well-being, the mediator variables were perceived defeat and internal entrapment, and the outcome variable was self-harm thoughts. The analysis controlled for gender and the presence of depressive symptomology on both the outcomes and mediators. This analysis was repeated, replacing internal entrapment with external entrapment. As both subtypes of entrapment are highly correlated, where internal entrapment was entered as a mediator, we included external entrapment as a covariate and vice versa. Direct and indirect effects were calculated for both models. Given that PROCESS expects complete data on all variables included in the model and that 7 participants (1.3%) had missing data, these analyses were conducted using 98.7% (*n* = 566) of the included sample. Missing data were handled using listwise deletion. Given that gender and severity of depressive symptomology have been shown to be robust predictors of self-harm, these were included as covariates within all analyses.

Finally, using Model 1 in PROCESS, a moderation model was tested whereby mental well-being at T1 moderates the relationship between entrapment (internal and external) at baseline and prospective self-harm thoughts. As with the previous analyses, when investigating one subscale of entrapment as a predictor, the other was included as a covariate, alongside depression and gender. While data were collected from individual pupils who were nested within their respective schools, it was determined that multilevel modelling analysis would not yield different results from non-multilevel techniques. This decision was supported by the fact that participants’ school did not significantly predict self-harm and that intraclass correlation coefficients suggested that there was no relationship between observations within schools.

## 3. Results

### 3.1. Preliminary Results

Of the young people who took part at baseline, 28.5% (*n* = 298) endorsed having ever thought about harming themselves. Within this subgroup, 37.6% (*n* = 112) had thought about engaging in self-harm, but had never acted on those thoughts, while 62.4% (*n* = 186) reported that their thoughts had progressed to actions. Of the adolescents who completed the survey at both time points, 16.2% (*n* = 92) had thought about self-harm during the six-month follow-up period. All these young people had reported a lifetime history of self-harm thoughts at T1. Exactly 50% (*n* = 46) of the adolescents who had considered harming themselves during the prospective period had acted on these thoughts. Of the young people who took part at baseline, 17.8% (*n* = 186) endorsed having ever engaged in self-harm. Of the adolescents who completed self-harm measures at both time points, 8.1% (*n* = 46) reported having engaged in self-harm during the six-month follow-up period. The majority (67.3%) of these young people described repetition of self-harm since T1, while the remaining 32.7% endorsed having engaged in self-harm for the first time between baseline and follow-up. Descriptive statistics (for all continuous variables) and correlational analyses (for all study variables) are presented in Table 1. The relationships between mental well-being and all other study variables were negative (all *p*-values < 0.001). Associations between depressive symptomology, defeat, internal entrapment, external entrapment, self-harm thoughts and self-harm behaviors were positive and statistically significant (all *p*-values < 0.001).

### 3.2. Does Mental Well-Being Protect Against Subsequent Self-Harm Thoughts and Behaviors?

Two logistic regression tests were applied to address the first aim of the study. Descriptive statistics demonstrated the perceptions of mental well-being were higher in those who had not thought about self-harm or engaged in self-harm during the follow-up period. The results of these logistic regressions demonstrated that young people who reported greater mental well-being at T1 were less likely report having thought about harming themselves (OR: 0.876, 95% CI: 0.820,.936, *p <* 0.001) or engaging in self-harm (OR: 0.913, 95% CI: 0.838,.995, *p =* 0.032) during the subsequent six month period.

### 3.3. Is Mental Well-Being Associated with Reduced Perceptions of Defeat and Entrapment?

A linear regression was applied to test the associations between positive well-being and each mediator (defeat, internal entrapment and external entrapment), resulting in three regressions in total. As both gender and depressive symptoms were found to predict perceptions of defeat, internal entrapment and external entrapment (all ps < 0.001), they were included as covariates in all regression models. Consistent with the second hypothesis, there was a significant negative association between mental well-being scores at T1 and participants’ perceptions defeat at T2 (R2 = 0.21, 0.001; *β* = −0.20, (*p* < 0.001) and external entrapment at T2 (R2 = 0.15, F(2, 566) = 50.02, *p <* 0.001; *β =* −0.19, *p <* 0.001). These relationships were independent of both covariates.

### 3.4. Do Perceptions of Defeat and Entrapment Mediate the Relationship Between Mental Well-Being and Subsequent Self-Harm Thoughts and Behavior?

**Figure 3:**
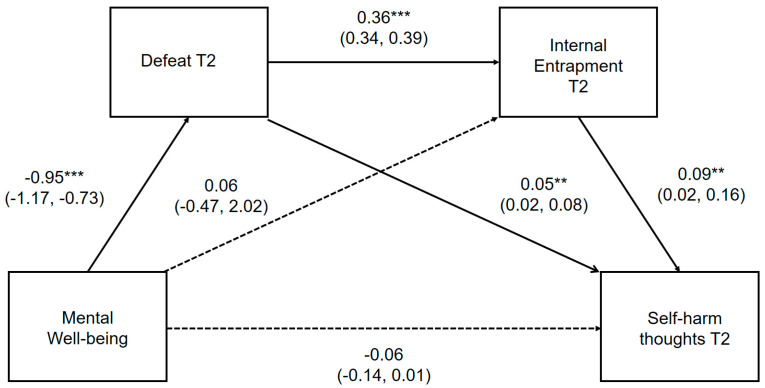
Serial multiple mediation model for the association between mental well-being and prospective self-harm thoughts, via defeat and internal entrapment. ** *p* < 0.01, *** *p* < 0.001 Serial multiple mediation model with unstandardized regression coefficients and 95% bias corrected confidence intervals. Significant pathways highlighted in bold.

**Figure 4:**
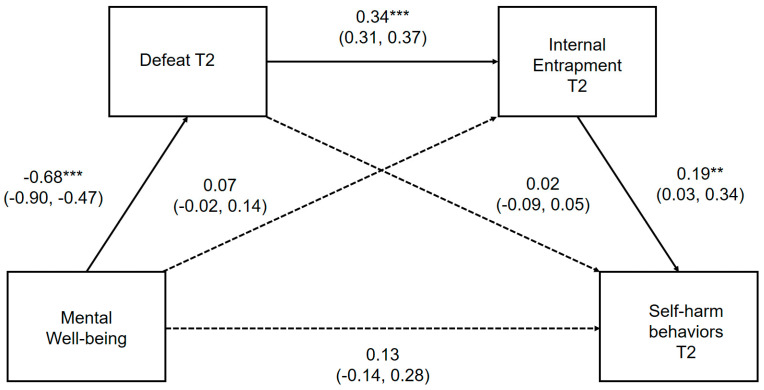
Serial multiple mediation model for the association between mental well-being and prospective self-harm behaviors, via defeat and internal entrapment. ** *p* < 0.01, *** *p* < 0.001 Serial multiple mediation model with unstandardized regression coefficients and 95% bias corrected confidence intervals. Significant pathways highlighted in bold.

The direct pathways between positive mental well-being, defeat, internal entrapment and self-harm thoughts and behaviors, controlling for gender, depression and external entrapment, are presented in Figure 3 and Figure 4, respectively. Where the outcome was self-harm behaviors, prospective thoughts of self-harm were included as a covariate. Unstandardized point estimates and bootstrapped 95% CIs for the total indirect effect and three specific indirect pathways are provided in Tables S1 and S2, which can be found in the supplementary materials.

Mental well-being was significantly associated with prospective self-harm thoughts and behaviors indirectly through defeat and internal entrapment assessed at T2. Perceptions of mental well-being, defeat and internal entrapment explained a moderate amount of the variability in the likelihood of reporting self-harm thoughts (pseudo R^2^; Cox and Snell = 0.20, Nagelkerke = 0.35; McFadden = 0.26) and behaviors (pseudo R^2^; Cox and Snell = 0.33, Nagelkerke = 0.74; McFadden = 0.68). Mental well-being did not maintain a significant direct relationship *(p =* 0.075) with future self-harm thoughts or behaviors *(p =* 0.063) within the full mediational model, demonstrating that reduced perceptions of defeat and internal entrapment account for the negative association between mental well-being and prospective self-harm thoughts and behaviors.

**Figure 5:**
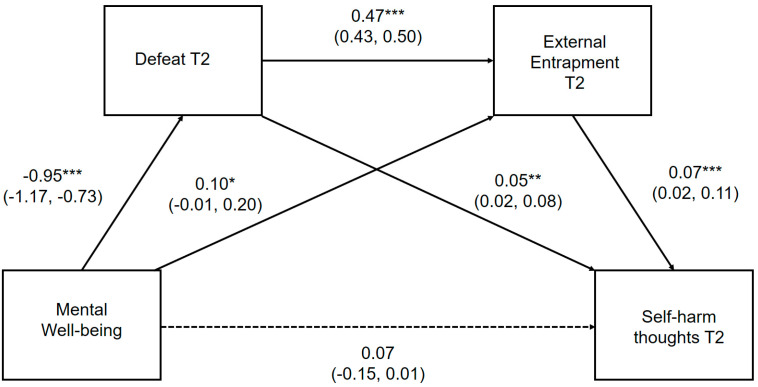
Serial multiple mediation model for the association between mental well-being and prospective self-harm thoughts, via defeat and external entrapment, * *p* < 0.05; *** *p* < 0.001 Serial multiple mediation model with unstandardized regression coefficients and 95% bias corrected confidence intervals. Significant pathways are highlighted in bold.

**Figure 6:**
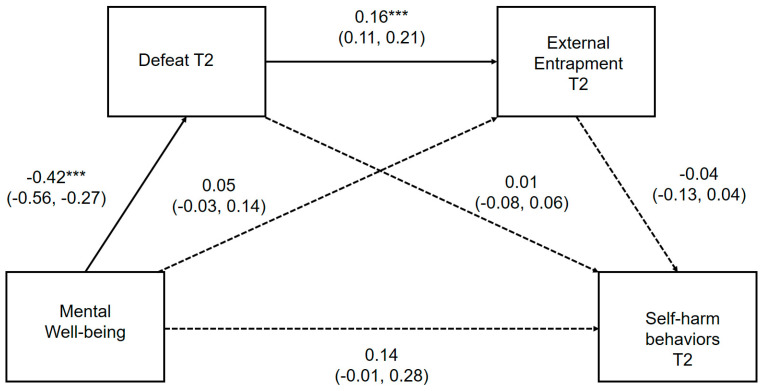
Serial multiple mediation model for the association between mental well-being and prospective self-harm behaviors, via defeat and external entrapment. *** *p* < 0.001 Serial multiple mediation model with unstandardized regression coefficients and 95% bias corrected confidence intervals. Significant pathways are highlighted in bold.

The direct pathways between mental well-being, defeat, external entrapment and self-harm thoughts and behaviors, controlling for gender and depression, are presented in Figure 5 and Figure 6, respectively. Where the outcome was self-harm behaviors, prospective thoughts of self-harm were included as a covariate. Unstandardized point estimates and bootstrapped 95% CIs for the total indirect effect and three specific indirect pathways are provided in Tables S3 and S4, which can be found in the supplementary materials.

Positive mental well-being was significantly associated with subsequent self-harm thoughts indirectly through defeat and external entrapment assessed at T2. Mental well-being, defeat and external entrapment explained a moderate amount of the variability in the likelihood of reporting self-harm thoughts (pseudo R^2^; Cox and Snell = 0.21, Nagelkerke = 0.35; McFadden = 0.26). Mental well-being did not maintain a significant direct relationship *(p =* 0.052) with future self-harm thoughts within the full mediational model, demonstrating that reduced perceptions of defeat and external entrapment account for the negative association between mental well-being and prospective thoughts of self-harm.

The hypothesized serial multiple mediation model from mental well-being to prospective self-harm behaviors via defeat and external entrapment, was not statistically significant *(β =* 0.001, 95% CI: −0.064, 0.155) when adjusting for gender, depressive symptomology, internal entrapment and self-harm thoughts assessed at T2. This model is significant when it does not control for prospective self-harm thoughts. Further examination of individual pathways suggest that prospective self-harm may fully mediate the relationship between entrapment and subsequent self-harm behaviors.

### 3.5. Does Mental Well-Being Moderate the Relationship Between Entrapment and Prospective Self-Harm Thoughts?

The overall model was not statistically significant for both internal *(p =* 0.482) and external entrapment *(p =* 0.493). These results suggest that mental well-being does not moderate the relationship between the internal/external entrapment and subsequent self-harm thought. The statistical estimates for the individual paths characterizing this model are provided in Tables S5 and S6, which can be found within the supplementary materials.

## 4. Discussion

The current study is the first to investigate the relationship between mental well-being and both self-harm thoughts and behaviors using a prospective research design. In addition, it is the first to test predictions derived from the IMV to advance understanding regarding the psychological mechanisms underpinning this link. The aims of this investigation were fourfold. First, we sought to examine whether mental well-being protects against future self-harm thoughts and behaviors. Second, we set out to determine if mental well-being was associated with perceptions of defeat and both internal and external entrapment. Third, we wished to test hypothesized multi-step pathways linking mental well-being and self-harm thoughts and behaviors, via perceptions of defeat and entrapment (internal and/or external). Fourth, we wished to determine if mental well-being moderated the relationship between entrapment and subsequent self-harm thoughts.

### 4.1. Mental Well-Being in Relation to Subsequent Self-Harm Thoughts and Behaviors

The current study extends the literature by examining mental well-being in relation to future self-harm thoughts and acts of self-harm during adolescence. Results demonstrated that young people with better mental well-being were less likely to report thinking about or engaging in self-harm during the six-month follow-up period. Importantly, these relationships persisted when controlling for gender and depressive symptomology. The findings thus support our hypothesis and are in line with cross-sectional research investigating this link in young people in the UK [35].

### 4.2. Mechanisms Linking Mental Well-Being and Self-Harm Risk: The Role of Defeat and Entrapment

Defeat and entrapment are robust and proximal predictors of self-harm. Our results highlight that young people with better mental well-being are more likely to report lower perceptions of subsequent defeat and entrapment. These findings support our second hypothesis and are consistent across both entrapment subscales (i.e., internal and external). This is the first study to investigate these self-harm related constructs in relation to mental well-being. Following on from that, the current study supports the hypothesized multi-step mediational pathway from mental well-being to self-harm thoughts. Specifically, young people who report better mental well-being are less likely to feel defeated, as a result of reduced defeat these younger people are less likely to feel trapped (either by their own thoughts and feelings or by their life circumstances). Reduced perceptions of entrapment are then associated with a reduced likelihood of thinking about self-harm in the future. Our work offers novel insights regarding the link between mental well-being and self-harm thoughts and reinforce the importance of defeat and entrapment as potentially transdiagnostic psychological constructs underlying self-harm thoughts and behaviors [48,49,50,51]. This is particularly interesting as no research has scientifically tested the relationship between mental well-being and defeat and entrapment in young people. These findings suggest that enhancing protective factors such as mental well-being may reduce young people’s experience of proximal risk factors for self-harm.

It is important to note that the negative relationship between mental well-being and internal entrapment was fully mediated by reduced perceptions of defeat, while lower levels of defeat only partially accounted for the link between mental well-being and reduced external entrapment. This suggests that additional pathways may underlie the latter relationship and that future research should focus on identifying these other candidate mechanisms. Further, these findings reinforce the value of looking at separate subscales of entrapment with regards to obtaining a refined understanding of how self-harm risk can be prevented or reduced in young people [39].

The current study supports the hypothesized multi-step mediational pathway from mental well-being to self-harm behaviors via defeat and internal entrapment when controlling for the experience of prospective thoughts of self-harm. Conversely our findings did not support the hypothesized multi-step model from mental well-being to self-harm behaviors via defeat and external entrapment. Further examination of the individual pathways supported a link between mental well-being and defeat and the relationship between defeat and external entrapment. The perception of feeling trapped by life circumstances (i.e., external entrapment) mediates the link between defeat and self-harm behaviors when prospective self-harm thoughts is not included as a covariate but disappears when this variable is adjusted for. Taken together, these findings suggest that subsequent thoughts of self-harm, assessed at follow-up, may mediate the relationship between external entrapment and prospective acts of self-harm. While it would have been advantageous to confirm this by examining the hypothesized model in full (i.e., mental well-being > defeat > entrapment > self-harm harm thoughts > self-harm behaviors) available software does not currently support the inclusion and testing of binary mediators within serial multiple mediation models. This is a limitation of our work and future research should seek to examine this pathway.

### 4.3. Establishing the Nature of the Link Between Mental Well-Being and Defeat

Within the current investigation, experiencing better mental well-being was strongly related to reduced perceptions of defeat. However, it is not yet clear why this is the case. As a result, research that seeks to uncover the nature of this link is warranted. What we do know is that mental well-being affords resilience to stressful events [52]. People who are characterized as being resilient can effectively cope with heightened stress and positively adapt to situations despite their experiences of significant adversity or trauma [53]. However, resilience does not eradicate stress or remove life adversities, instead it gives people the tools to handle problems effectively and overcome adversity [54]. As such, mental well-being may increase young people’s resilience and act as a buffer against the impact of stress by providing young people with the resources to “bounce back” from difficult experiences [55]. These resources may come from feeling good and functioning well at the individual level (e.g., optimism and the ability to think clearly) or in affiliation with others by forming strong and supportive interpersonal relationships. In turn, these young people may feel less defeated in response to stressful circumstances. Future research could look at the aspects of mental well-being (e.g., optimism, feeling useful, feeling close to others) that are most influential within this context.

### 4.4. Mental Well-Being as a Moderator of the Relationship Between Entrapment and Self-Harm Thoughts

Contrary to our hypothesis and preliminary evidence in adults, our findings demonstrate that mental well-being did not moderate the relationship between entrapment (internal and external) and self-harmful thoughts as has been found elsewhere in the literature [34]. Several methodological differences between the current investigation and the work of Teisman et al. [34] may explain these different findings. First, our sample comprised young people in the community, while previous work focused on a substantially wider age range (18–77 years old). Adolescence is a unique developmental period and so it may be that this finding does not apply to young people. Second, we focused on self-harm more broadly, while the previous investigation assessed thoughts specifically associated with suicidal intent. Third, our research was prospective and so it may be that these findings only apply cross-sectionally. Fourth, there were differences in measurement of mental well-being between the current investigation and the work of Teisman et al. [34]. While we employed the SEWMWBS, Teisman et al. [34] assessed mental well-being using the psychological well-being scale, which has six separate subscales (e.g., autonomy, environmental mastery, purpose in life). While there is some overlap (i.e., both consider positive relationships with others), it could be argued that they are tapping into different aspects of mental well-being. These studies are the first to examine this relationship and so further replication is necessary.

### 4.5. Strengths and Limitations

The current study has three key strengths. First, the prospective design of the current investigation provides novel insights into the role of mental well-being as a protective factor for future thoughts and self-harm. By collecting longitudinal data, we were able to take a positive step beyond examining mental well-being as a correlate of these thoughts and behaviors, which is valuable in understanding the role of mental well-being in self-harm. Second, the current investigation employed standardized measures that were validated for use within adolescent samples [23,45]. Third, the current investigation recruited a sample of adolescents from the community. Research has consistently demonstrated that most of the adolescent self-harm occurs “hidden” in the community and does not come to the attention of clinical services [3,56]. The hidden nature of most self-harm, alongside the reported differences between young people who do and do not present to health services after harming themselves [57,58], highlights the need for community-based research and prevention efforts.

Despite these strengths, findings should be interpreted within the context of the following two limitations. First, and most notably, attrition was high between waves of data collection, and one school withdrew their participation entirely. The follow-up response rate of the current investigation was 54.8%, which is lower than that of another longitudinal study of self-harm in Scotland (69.8%) [40]. As such, it is possible that this attrition may have biased our results in terms of prevalence estimates and associations. Though adolescents who took part in the second wave of data collection had broadly similar profiles across most variables compared with those who did not, it was also true that those lost to attrition were more likely to report a history of self-harm at baseline. This has been reported in previous research [59] and is not surprising given that young people who engage in self-harm are more likely to be absent or truant from school [60]. Therefore, we were less likely to capture these individuals at follow-up. As our study is likely to have underestimated the number of participants that engaged in repeat self-harm, our findings should be interpreted with caution.

While the current investigation is prospective in nature, there were only two waves of data collection. As a result of overlapping time periods in the assessment of some variables, it was not possible to determine temporal precedence with regards the multistep mediational pathway linking mental well-being and self-harm thoughts [61]. Therefore, it is not possible to rule out alternative causal pathways. Future research applying multi-wave longitudinal cohort designs, would allow for an enhanced understanding of how processes unfold over time during adolescence. Further, micro-longitudinal investigations, such as daily diary studies or work implementing experience sampling methodologies, would allow for a more high-resolution examination of the relationship between these variables in daily life.

### 4.6. Implications

This investigation provides preliminary evidence that mental well-being is a factor that should be included within the pre-motivational phase of the IMV, conceptualized as a *protective* background factor. Public mental health encompasses the promotion of mental well-being, the prevention of mental illness and recovery from mental health problems. Most research to date has focused on the role of mental health problems in relation to self-harm risk during adolescence. While this research does improve our understanding of the challenges that young people experience (and how these may heighten a young person’s vulnerability to thinking about or engaging in self-harm), the current study demonstrates the value of considering other dimensions of public mental health and investigating factors that may reduce self-harm risk. Further research of this nature will provide novel insights that could help to refine theoretical conceptualizations of self-harm.

The current investigation is the first to examine the link between mental being and adolescent self-harm risk within the context of a theoretical framework. Our findings suggest that greater mental well-being is associated with lower risk of self-harm thoughts and behaviors, as well as a reduction in defeat and entrapment (the negative appraisals that have been shown to be proximal predictors of intention to harm themselves). These results highlight that mental well-being could be one useful target which, if incorporated within prevention and early intervention efforts, could protect young people against self-harm risk.

Mental well-being is modifiable, and schools are a natural setting for the implementation of programmes that seek to maintain or improve mental well-being during adolescence. Taking a universal approach (and including all young people in a year group or school) can potentially reduce stigma and is particularly suited to a focus on mental well-being [62]. There is preliminary evidence to suggest that programmes of this nature can increase mental well-being in young people. These include teaching mindfulness as a way of working with every day stressors and experiences or accessing websites such as “bite back” that consists of information and interactive activities that relate to a variety of different well-being domains including gratitude, optimism, healthy lifestyle and positive relationships [63]. Nature-based prescribing may also have the potential to support and improve mental well-being and could be considered alongside school-based programmes [64]. Taken together, findings supporting the amenability of mental well-being to intervention are particularly important given its protective role within the context of self-harm risk and wider public health.

## 5. Conclusions

Previous research has largely focused on factors that heighten a young person’s vulnerability to self-harm risk and investigations focusing on protective factors are limited. Research of this nature is crucial if we are to better understand and reduce self-harm within the adolescent population. Mental well-being is receiving increasing attention worldwide, and our findings show that it may offer protection against self-harm risk by reducing perceptions of defeat and entrapment. Future prevention and intervention efforts should incorporate strategies that promote mental well-being.

## Figures and Tables

**Table 1:** Means, standard deviations and Spearman correlational coefficients for mental well-being, depression, defeat, internal entrapment, external entrapment and self-harm thoughts and behaviors.

Variable/Metric	1	2	3	4	5	6	7
1. Mental well-being							
2. Depressive symptoms	−0.51 ***						
3. Defeat	−0.52 ***	0.41 ***					
4. Internal entrapment	−0.42 ***	0.38 ***	0.78 ***				
5. External entrapment	−0.37 ***	0.34 ***	0.76 ***	0.84 ***			
6. SHT T2	−0.27 ***	0.19 ***	0.42 ***	0.45 ***	0.43 ***		
7. SHB T2	−0.16 ***	0.11 **	0.32 ***	0.35 ***	0.34 ***	0.67 ***	
*Median*	22.85	3.00	12.00	1.50	2.00	-	-
*Interquartile range*	5.78	4.00	17.00	6.00	7.00	-	-

Variables 2 to 7 within the correlation matrix collected at the 6-month follow-up, SHT T2—self-harm thoughts during 6 month follow-up. SHB T2—self-harm behaviors during 6-month follow up *** *p* < 0.001, ** *p* < 0.01. Where variables are dichotomous the symbol (-) indicated that it was not possible to calculate a median and interquartile range.

## Supplementary Materials

The following are available online at https://www.mdpi.com/1660-4601/17/18/6771/s1, Table S1, Point estimates for indirect effects and 95% bias corrected confidence intervals for serial multiple mediation analysis in which defeat and internal entrapment were represented as mediators in the association mental well-being and self-harm thoughts (controlling for gender, depression and external entrapment); Table S2, Point estimates for indirect effects and 95% bias corrected confidence intervals for serial multiple mediation analysis in which defeat and external entrapment were represented as mediators in the association between mental well-being and self-harm thoughts (controlling for gender, depression and internal entrapment); Table S3, Point estimates for indirect effects and 95% bias corrected confidence intervals for serial mualtiple mediation analysis in which defeat and internal entrapment were represented as mediators in the association mental well-being and self-harm behaviors (controlling for gender, depression and external entrapment); Table S4, Point estimates for indirect effects and 95% bias corrected confidence intervals for serial multiple mediation analysis in which defeat and external entrapment were represented as mediators in the association between mental well-being and self-harm behaviors (controlling for gender, depression and internal entrapment); Table S5, Point estimates for effects and 95% confidence intervals for moderation analysis in which mental well-being was represented as a moderator in the relationship between internal entrapment at baseline and prospective self-harm thoughts (controlling for gender, depression and external entrapment); Table S6, Point estimates for effects and 95% confidence intervals for moderation analysis in which mental well-being was represented as a moderator in the relationship between external entrapment at baseline and prospective self-harm thoughts (controlling for gender, depression and internal entrapment).
