# Supplementary material for: Does Mental Well-Being Protect against Self-Harm Thoughts and Behaviors during Adolescence? A Six-Month Prospective Investigation
Source: Int J Environ Res Public Health. 2020 Sep 17;17(18):6771. doi: 10.3390/ijerph17186771 (PMC7558682; doi:10.3390/ijerph17186771)
Supplement: Supplementary file 1 [file ijerph-17-06771-s001.pdf]

International Journal of
Environmental Research and Public Health

MDPI

Table S1. Point estimates for indirect effects and 95% bias corrected confidence intervals for serial multiple mediation analysis in which defeat, and internal entrapment were represented as mediators in the association mental well-being and self-harm thoughts (controlling for gender, depression, and external entrapment).

	95% Confidence Intervals [a]		
**Mediation Path**	**Point Estimate**	**Lower**	**Upper**
**Mental well-being > Defeat > SHT**	−0.05	−0.09	−0.02
**Mental well-being > Internal Entrapment > SHT**	0.01	−0.01	0.01
**Mental well-being > Defeat > Internal Entrapment > SHT**	−0.03	−0.06	−0.01
**Total indirect effect**	0.07	−0.10	−0.05

a Statistical significance indicated by confidence intervals not containing 0; SHT—self-harm.

Table S2. Point estimates for indirect effects and 95% bias corrected confidence intervals for serial multiple mediation analysis in which defeat, and external entrapment were represented as mediators in the association between mental well-being and self-harm thoughts (controlling for gender, depression, and internal entrapment).

	95% Confidence Intervals [a]		
**Mediation Path**	**Point Estimate**	**Lower**	**Upper**
**Mental well-being > Defeat > SHT**	−0.05	−0.09	0.05
**Mental well-being > External Entrapment > SHT**	0.01	0.01	0.02
**Mental well-being > Defeat > External Entrapment > SH**	−0.03	−0.06	−0.01
**Total indirect effect**	0.07	−0.10	−0.05

a Statistical significance indicated by confidence intervals not containing 0; SHT—self-harm thoughts

Table S3. Point estimates for indirect effects and 95% bias corrected confidence intervals for serial multiple mediation analysis in which defeat, and internal entrapment were represented as mediators in the association mental well-being and self-harm behaviors (controlling for gender, depression, and external entrapment).

	95% Confidence Intervals [a]		
**Mediation Path**	**Point Estimate**	**Lower**	**Upper**
**Mental well-being > Defeat > SHB**	0.01	−0.04	0.09
**Mental well-being > Internal Entrapment > SHB**	0.01	−0.00	0.05
**Mental well-being > Defeat > Internal Entrapment > SHB**	−0.04	−0.12	−0.01
**Total indirect effect**	−0.02	−0.06	0.03

a Statistical significance indicated by confidence intervals not containing 0; SHB—self-harm behavior.

Table S4. Point estimates for indirect effects and 95% bias corrected confidence intervals for serial multiple mediation analysis in which defeat, and external entrapment were represented as mediators in the association between mental well-being and self-harm behaviors (controlling for gender, depression, and internal entrapment).

Mediation Path	Point Estimate	95% Confidence Intervals [a] Lower	Upper
**Mental well-being > Defeat > SHT**	0.004	−0.026	0.055
**Mental well-being > External Entrapment > SHB**	−0.002	−0.012	0.005
**Mental well-being > Defeat > External Entrapment > SHB**	0.003	−0.003	0.012
**Total indirect effect**	0.005	−0.025	0.057

a Statistical significance indicated by confidence intervals not containing 0; SHT—self-harm thoughts; SHB—self-harm behavior.

Table S5. Point estimates for effects and 95% confidence intervals for moderation analysis in which mental well-being was represented as a moderator in the relationship between internal entrapment at baseline and prospective self-harm thoughts (controlling for gender, depression, and external entrapment).

Moderations Path	β	*p*	95%CI
Internal Entrapment → SHT	0.31	0.005	0.09, 0.53
Internal Entrapment x Mental Well-being → SHT	−0.01	0.165	−0.02, 0.01

SHT—self-harm thoughts.

Table S6. Point estimates for effects and 95% confidence intervals for moderation analysis in which mental well-being was represented as a moderator in the relationship between external entrapment at baseline and prospective self-harm thoughts (controlling for gender, depression, and internal entrapment).

Moderation Path	β	*p*	95%CI
External Entrapment → SHT	0.18	.014	0.36, 0.33
External Entrapment x Mental Well-being → SHT	−0.01	0.211	−0.01, 0.00

SHT—self-harm thoughts.

© 2020 by the authors. Licensee MDPI, Basel, Switzerland. This article is an open access article distributed under the terms and conditions of the Creative Commons Attribution (CC BY) license (http://creativecommons.org/licenses/by/4.0/).
